# Unilateral Osler nodes, Janeway lesions and splinter haemorrhages associated with surgical arterio-venous fistula infection: a case report

**DOI:** 10.1186/s12879-023-08439-x

**Published:** 2023-07-06

**Authors:** Pramith Ruwanpathirana, Harindri Athukorala, Praveen Weeratunga, Panduka Karunanayake

**Affiliations:** 1 Professorial Unit in Medicine, National Hospital of Sri Lanka, Colombo, 01000 Sri Lanka; 2 Department of Clinical Medicine, University of Colombo, 25, Kynsey Road, Colombo, 00800 Sri Lanka

**Keywords:** Osler’s nodes, Janeway lesions, Splinter haemorrhages, Arterio-venous fistula, Haemodialysis

## Abstract

**Background:**

Osler’s nodes, Janeway lesions and splinter haemorrhages are cutaneous manifestations of infective endocarditis. They occur due to vascular occlusion by septic emboli and a resulting localized vasculitis. They are usually bilateral. We report a case of unilateral Osler’s nodes, Janeway lesions and splinter haemorrhages due to an ipsilateral surgical arterio-venous fistula infection.

**Case presentation:**

A fifty-two-year-old Sri Lankan female with end stage kidney disease presented with fever for five days with blurred vision, pain and redness of the right eye. She had a left brachio-cephalic arterio-venous fistula (AVF) created one month back. She complained of a foul-smelling discharge from the surgical site for past three days. Redness of the right eye with a hypopyon was noted. AVF site over the left cubital fossa was infected with a purulent discharge. Osler’s nodes, Janeway lesions and splinter haemorrhages were noted in the distal fingers, thenar and hypothenar eminences of the left hand. Right hand and both feet were normal. No cardiac murmurs were heard. Blood cultures, vitreous sample cultures and pus cultures from the fistula site were all positive for methicillin sensitive *Staphylococcus aureus*. Infective endocarditis was excluded by a trans-oesophageal echocardiogram. She was treated with IV flucloxacillin and surgical excision of the AVF.

**Conclusion:**

Infections of AVF can result in septic emboli formation which can have both anterograde arterial embolization and retrograde venous embolization. Arterial embolization can result in unilateral Osler’s nodes, Janeway lesions and splinter haemorrhages. Venous embolization can cause metastatic infections in the systemic and pulmonary circulations.

## Introduction

Osler’s nodes, Janeway lesions and splinter haemorrhages are cutaneous manifestations of infective endocarditis [1]. Osler’s nodes are tender violaceous cutaneous nodules that occur in the distal ends of fingers and over thenar and hypothenar eminences. It is hypothesized to occur due to vascular occlusion by septic micro-emboli causing localised vasculitis with or without micro-abscess formation [2]. Janeway lesions are non-tender, erythematous macules commonly found on palms and soles [3]. Splinter haemorrhages are red–brown to purple–black, thin longitudinal lines in the distal portion of nails which occur in diverse conditions including infective endocarditis [4]. The above cutaneous signs usually occur bilaterally. We describe a case of unilateral Osler’s nodes, Janeway lesions and splinter haemorrhages in the setting of an infected ipsilateral brachio-cephalic arterio-venous fistula (AVF).

## Case presentation

A fifty-two-year-old Sri Lankan female with end stage kidney got admitted with five days of fever and a painful red right eye. She was receiving regular haemodialysis via a right subclavian tunnelled catheter. The chronic kidney disease was secondary to long standing, poorly controlled diabetes and hypertension. Her diabetes was complicated with proliferative retinopathy and right sided vitreous haemorrhage which occured 2 weeks ago. The visual acuity in the right eye following the haemorrhage was 20/70. Over the last five days the vision deteriorated to complete blindness. She had a left sided brachio-cephalic AVF created one month back. There was a purulent discharge from the surgical site over the past 3 days.

**Fig. 1:**
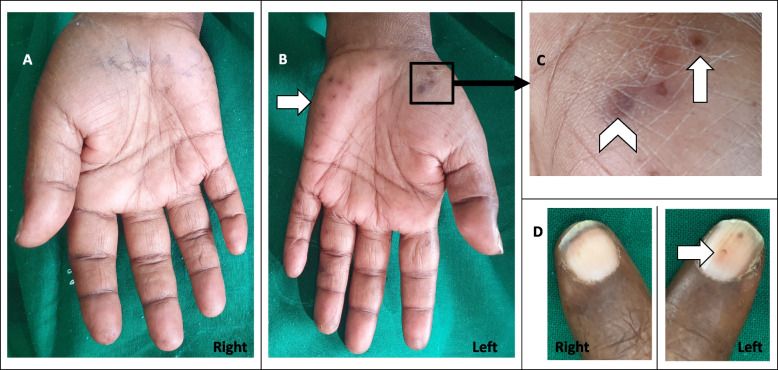
Right hand (**A**) is normal while the left hand (**B**) has Osler’s nodes and Janeway lesions over the thenar and hypothenar eminences (arrow). The lesions over the thenar eminence are magnified in **C** which shows Osler’s nodes (arrow) and Janeway lesions (arrowhead). Splinter haemorrhages of the left nailbed (arrow) and the normal nails of the right hand are shown in **D**

On examination she had a right sided red eye with a hypopyon, suggestive of endophthalmitis. The surgical site was dehiscent with foul smelling pus discharge. A thrill was felt at the fistula site. The left hand had Osler’s nodes, Janeway lesions and splinter haemorrhages (Fig. 1). The right hand and feet were normal. There were no cardiac murmurs.

Three blood cultures from different sites, vitreous sample cultures and surgical site swab cultures were persistently positive for methicillin sensitive *Staphylococcus aureus* (MSSA). The in-vitro incubation periods were similar for blood cultures from the peripheral veins and the tunnelled catheter. The tunnelled catheter was removed, and the tip culture did not grow any organisms. No valvular vegetations, insufficiency or abscesses were noted in the trans-oesophageal echocardiogram. There was no evidence of a patent foramen ovale. A diagnosis of ‘left brachio-cephalic AVF surgical site infection and right endophthalmitis due to systemic embolization’ was arrived at. The relevant biochemical and haematological parameters on admission are given in Table 1.

**Table 1:** Biochemical and haematological parameters on admission

Parameter	Value	Normal Range
White blood Cell Count (× 10^9^/L)	22	4—11
Neutrophil	84%	40 – 60
Lymphocyte	12%	20 – 40
ESR (mm/1^st^ hour)	110	< 30 (age adjusted)
C-Reactive Protein (mg/dL)	198	< 0.3
Serum Creatinine (mg/dl)	5.87	0.6 – 1.1
Serum Na^+^ (mmol/l)	138	136 – 145
Serum K^+^ (mmol/l)	5.0	3.5 – 5.5
ECG	Electrical criteria for left ventricular hypertrophy	
Chest X ray	Cardiomegaly with mild bilateral pleural effusions No features to suggest consolidations or lung abscess	

*ESR* Erythrocyte sedimentation rate, *ECG* Electrocardiogram

She was treated with high dose IV flucloxacillin for 4 weeks and intra vitreous vancomycin. A temporary vascular catheter was inserted to the right internal jugular vein after 3 days of IV antibiotics, by which time the fever had settled; for haemodialysis every third day. The temporary catheter was changed every 6^th^ day. The surgical site was explored, irrigated and the fistula was excised. The excised tissue did not grow any organisms; probably because the excision was done 1 week after the initiation of IV antibiotics. The infection was completely treated, but the patient continued to be blind in the right eye.

## Discussion and conclusions

We report a case of unilateral Osler’s nodes, Janeway lesions and splinter haemorrhages secondary to AVF site infection. Arterio-venous fistulae are created for easy vascular access during haemodialysis in patients with end-stage kidney disease. A brachio-cephalic fistula is created by anastomosing the brachial artery and the cephalic vein at the cubital fossa [5]. In a mature brachio-cephalic fistula, most of the blood in the brachial artery flow into the cephalic vein while the rest, flow to the forearm and hand along the radial and ulnar arteries.

The incidence of arterio-venous fistulae infection is around 0.9% [6]. Despite the common occurrence, unilateral Osler’s nodes, Janeway lesions and Splinter haemorrhages are not recognised signs of AVF infections. Mousa et al. [7] have described a patient with a left brachio-axillary polytetrafluoroethylene (PTFE) vascular graft infection, who was found to have splinter haemorrhages and Janeway lesions on the left hand without similar findings in the right hand. This probably is the first reported case of unilateral Janeway lesions or splinter haemorrhages [7]. Despite an extensive search we could not find any other case describing the above phenomenon. We report the first case of direct surgical arterio-venous fistula (without a prosthetic graft) infection leading to unilateral Janeway lesions, Osler’s nodes and splinter haemorrhages.

**Fig. 2:**
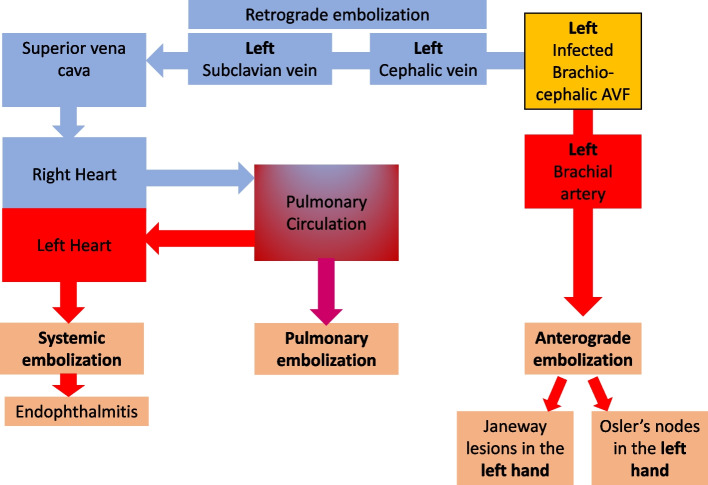
Pathways taken by septic emboli in anterograde arterial embolization and retrograde venous embolization

Infection of the AVF can be either a peri-vascular cellulitis or infection of the vessels itself (endarteritis) [8]. We hypothesize that the infection of the brachio-cephalic fistula generated septic micro-thrombi which embolized both distally along the radial and ulnar arteries, and proximally along the arterialised cephalic vein. The fistula provided a means to bypass the capillary circulation which would otherwise filter the emboli, preventing it from reaching the venous circulation. Anterograde (distal), localised brachial arterial embolization resulted in Osler’s nodes, Janeway lesions and splinter haemorrhages in the ipsilateral hand whereas retrograde/venous embolization led to systemic (and potentially pulmonary) embolization. In the index patient, systemic embolization manifested as right side endophthalmitis. The pathways taken by arterial and venous emboli are depicted in Fig. 2.

Despite the fact that AVF site infection cause retrograde-venous embolization which reach the systemic circulation, Osler’s nodes and Janeway lesions were not noted on the right hand or feet in the index patient. Although there is a high blood flow from the brachial artery to the cephalic vein (due to the high-pressure gradient between the artery and the vein), we hypothesize that the retrograde septic emboli got filtered in the pulmonary circulation. Therefore, blood flowing to the contralateral (right) hand would have had a lesser concentration of emboli leading to a lesser density of embolic phenomena (or none), compared to anterograde brachial arterial embolization to the ipsilateral (left) hand. Furthermore, the venous emboli would have been diluted in a higher volume of blood at the cardiac chambers. This also would contribute to the difference in the number of Osler’s nodes and Janeway lesions between the two hands.

Therefore, we can postulate that; in brachio-cephalic AVF infections, the hand ipsilateral to the AVF (with anterograde arterial embolization) will have a higher density of embolic phenomena whereas the opposite hand and legs (embolization through systemic circulation) will have a comparably lower number of lesions; or none as in the index case.

In conclusion AVF infections can cause both arterial and venous embolization. Arterial embolization can manifest as unilateral (or differential) Osler’s nodes, Janeway lesions and splinter haemorrhages of the ipsilateral hand. Venous embolization can lead to metastatic infection in the systemic and pulmonary circulations.

## Data Availability

The clinical records of the patient are available from the corresponding author on reasonable request.

## Abbreviations

AVF: Arterio-venous fistula
ECG: Electrocardiogram
ESR: Erythrocyte sedimentation rate
MSSA: Methicillin sensitive *Staphylococcus aureus*
PTFE: Polytetrafluoroethylene
